# Supplementary material for: Urine Protein to Creatinine Ratio for the Assessment of Bevacizumab-Associated Proteinuria in Patients with Gynecologic Cancers: A Diagnostic and Quality Improvement Study
Source: Diagnostics (Basel). 2024 Aug 24;14(17):1852. doi: 10.3390/diagnostics14171852 (PMC11394588; doi:10.3390/diagnostics14171852)
Supplement: Supplementary file 1 [file diagnostics-14-01852-s001.zip › diagnostics-3149779-supplementary.pdf]

Table S1. Common Terminology Criteria for Adverse Events 5.0 for proteinuria, Associated Tests and Their Parameters.

CTCAE grade	1	2	3	4
24-hour urine total protein (g)	< 1.0	1.0 – 3.5	≥ 3.5	Nephrotic syndrome
Simple urine dipstick (range, mg/dL)	30	100	300	1000
	1+	2+	3+	4+
UACR dipstick (range, mg/gCr) *	30 – 300	≧ 300		
	1+	2+		

Not included in CTCAE. CTCAE, Common Terminology Criteria for Adverse Events.

Table S2 Cost Analysis for UPCR 2.0 compared to Simple Urine Dipstick Based on Prevalence of Grade 2+ and More Proteinuria

Prevalence (%)	2.2	20	63
Estimated cost reduction in NTUH setting (%)	17.92	14.81	7.57
UPCR/urine dipstick *	1.42	1.35	1.20
ICER for reducing unnecessary 24-h urine sampling (USD/person)	0.37	0.43	0.67

Within the estimations, we assume that the costs for urine total protein and creatinine, regardless of a random urine sample or a 24-hour urine sample, are the same. And the cost for a UPCR is the sum of urine total protein and creatinine. *The ratio of the price for a UPCR test compared to a urine dipstick, when the total costs for the stepwise proteinuria assessment tests via urine dipstick and UPCR 2.0 are the same. ICER, incremental cost-effectiveness ratio.
